# MicroRNA-205 promotes hair regeneration by modulating mechanical properties of hair follicle stem cells

**DOI:** 10.1073/pnas.2220635120

**Published:** 2023-05-22

**Authors:** Jingjing Wang, Yuheng Fu, Wenmao Huang, Ritusree Biswas, Avinanda Banerjee, Joshua A. Broussard, Zhihai Zhao, Dongmei Wang, Glen Bjerke, Srikala Raghavan, Jie Yan, Kathleen J. Green, Rui Yi

**Affiliations:** ^a^Department of Pathology, Northwestern University Feinberg School of Medicine, Chicago, IL 60611; ^b^Department of Dermatology, Northwestern University Feinberg School of Medicine, Chicago, IL 60611; ^c^Mechanobiology Institute, National University of Singapore 117411, Singapore; ^d^Institute for Stem Cell Science and Regenerative Medicine, GKVK Campus, Bangalore 560065, India; ^e^A*Star Skin Research Institute of Singapore, Singapore 138648, Singapore; ^f^Robert H. Lurie Comprehensive Cancer Center, Northwestern University Feinberg School of Medicine, Chicago, IL 60611; ^g^Department of Molecular, Cellular and Developmental Biology, University of Colorado Boulder, Boulder, CO 80309

**Keywords:** cell mechanics, actomyosin contractility, hair follicle stem cells, hair regeneration, miRNA

## Abstract

Stiffness and actomyosin contractility are intrinsic mechanical properties of animal cells required for the shaping of tissues. However, whether tissue stem cells (SCs) and progenitors located within SC niche have different mechanical properties that modulate their size and function remains unclear. Here, we show that hair follicle SCs in the bulge are stiff with high actomyosin contractility and resistant to size change, whereas hair germ (HG) progenitors are soft and periodically enlarge and contract during quiescence. During activation of hair follicle growth, HGs reduce contraction and more frequently enlarge, a process that is associated with weakening of the actomyosin network, nuclear YAP accumulation, and cell cycle reentry. Induction of *miR-205*, a novel regulator of the actomyosin cytoskeleton, reduces actomyosin contractility and activates hair regeneration in young and old mice. This study reveals the control of tissue SC size and activities by spatiotemporally compartmentalized mechanical properties and demonstrates the possibility to stimulate tissue regeneration by fine-tuning cell mechanics.

## Significance

In this study, we have determined spatiotemporal compartmentalization of mechanical properties and their associated cell activities within the HF-SC niche during quiescence and activation. The spatial compartmentalization of stiff and quiescent hair follicle stem cells and soft and primed HGs is distinct from the architecture of the epidermis, where the basal, proliferative SC layer is soft and the suprabasal, differentiated layer is stiff. We have identified miR-205, a versatile and potent regulator of the actin cytoskeleton, whose expression modulates actomyosin contractility and the dynamics of cell size changes. Together, this study has implicated the dynamic changes of cell size and subsequent cell cycle reentry as a mechanism to sense and respond to mechanical forces during tissue regeneration.

Tissue regeneration is essential for homeostasis, wound repair, and aging. Adult stem cells (SCs) and their microenvironment control tissue regeneration by constantly sensing and responding to extrinsic and intrinsic cues ranging from signaling molecules to mechanical forces. The response of living cells to mechanical forces is modulated by physical properties of both external microenvironment, such as extracellular matrix (ECM) stiffness, and internal force-generating machinery, such as the cytoskeleton (1, 2). These mechanical properties have a profound impact on cell behaviors including cell adhesion, division, migration, and differentiation (1, 3, 4). However, although mechanical forces continuously regulate cellular and tissue functions, it has been challenging to directly observe their effects on cell size and activities of SCs within the SC niche during homeostasis and regeneration. It is unclear whether spatiotemporally demarcated SC activities, such as quiescence and cell cycle reentry, within distinct regions of the SC niche are regulated by local stiffness and differentially generated mechanical forces.

Mammalian skin is an excellent experimental system to interrogate the function of mechanical mechanisms on SC activity, tissue regeneration, wound repair, and tumorigenesis (5–10). A subset of epidermal SCs rapidly responds to external force-induced stretch by transiently activating cell division (5). Perturbations in the force-generating cytoskeleton and ECM through genetic deletion of key components of these machinery change mechanical properties of the cells (6, 8), and these changes compromise tissue functions. Interestingly, basal epithelial SCs and cultured keratinocytes have been shown to control their cell-cycle progression in a cell size-dependent manner (11, 12). However, whether cell mechanics controls cell size and regulates the transition between quiescence and activation of hair follicle stem cells (HF-SCs) and HG progenitors remains poorly understood. In this study, we uncovered spatiotemporally demarcated mechanical properties of tissue SCs and progenitors as tunable features to promote hair regeneration through the modulation of actomyosin contractility and cell size dynamics.

## Results

### HF-SC Compartment Is Demarcated by Differential Mechanical Stiffness and Actomyosin Contractility.

**Fig. 1.**
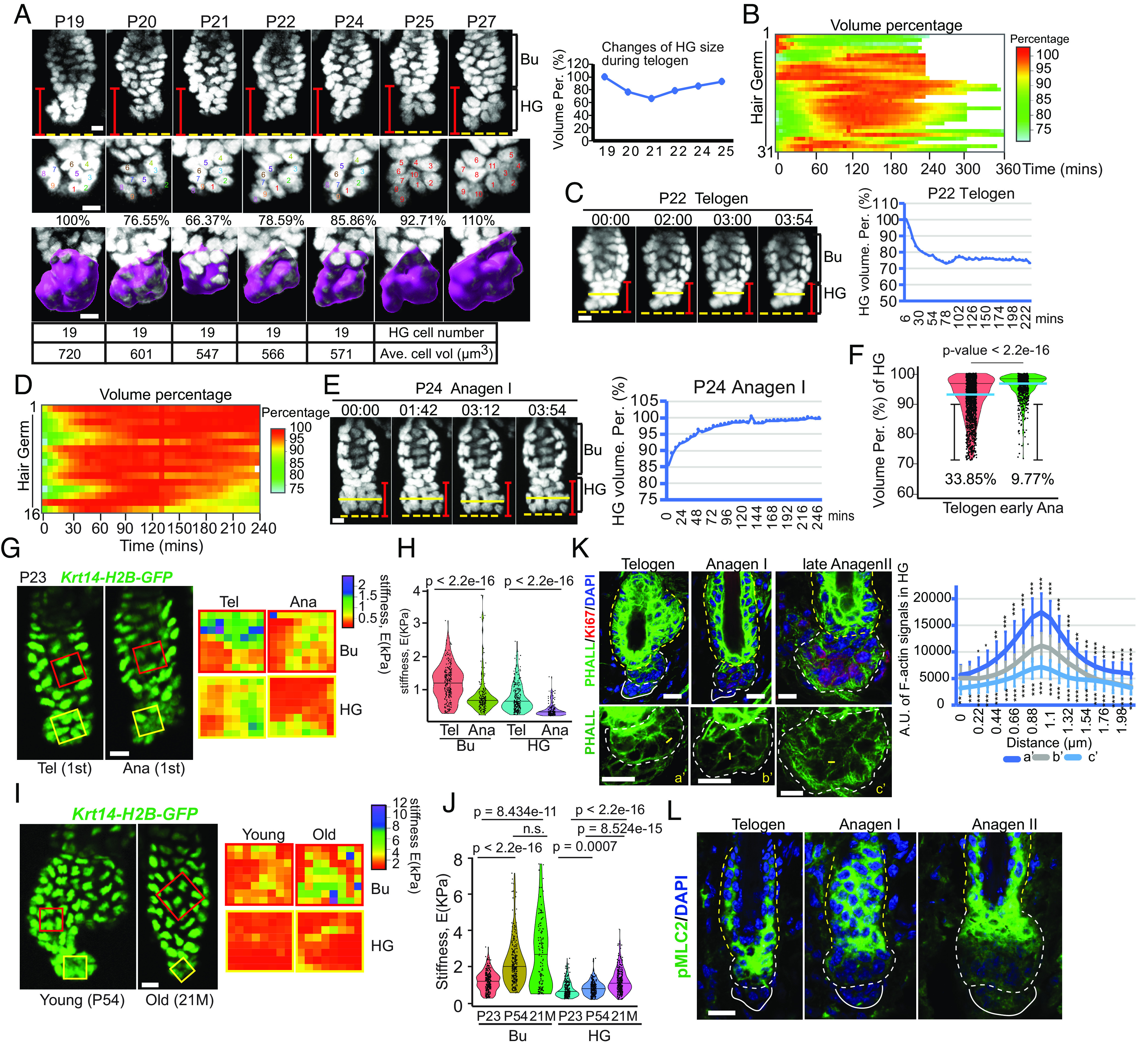
HF-SC compartment is mechanically active and exhibits differential mechanical stiffness and actomyosin contractility during activation. (*A*) Hair germ (HG) showed dynamic volume change in late catagen and telogen in the absence of cell division. Bu, bulge; HG, hair germ. Purple surfaces indicate HG areas used for quantification in Imaris. Totally 65 hair follicles from four animals were tracked. (*B*) HGs in telogen showed pulsatile contraction and enlargement. A total of 31 hair follicles from nine animals were used to calculate volume changes over 4 to 6 h. (*C*) An example of HG undergoing continuous contraction (*Left*) with quantification (*Right*) over 4 h in telogen. (*D*) HGs in early anagen with reduced contraction, compared to HGs in telogen. For quantification, 16 hair follicles from five animals were recorded. (*E*) An example of HG undergoing continuous enlargement (*Left*) with quantification (*Right*) over 4 h in early anagen without cell division. (*F*) HGs in early anagen showed reduced contraction, compared with those in telogen. The light blue lines represent median (med), and black lines in the violin plot represent the third quarter of all time points. (*G*) Stiffness of the bulge and HG in first telogen and anagen was measured ex vivo by atomic force microscopy (AFM). Force maps (with 64 measured points) of the bulge and HG are shown in the *Right* panel. (*H*) Quantification of stiffness (Young’s modulus) of bulge and HG in first telogen and anagen. Four hair follicles from two animals in telogen and anagen, respectively, were measured. For each area, 64 points were measured. In telogen, Bu mean = 1,196 ± 534 Pa, HG mean = 747.3 ± 414.3 Pa; in anagen, Bu mean = 798.3 ± 530.7 Pa, HG mean =374.9 ± 183 Pa. *P* values were determined by the Mann–Whitney *U* test. Number following the ± sign shows SD. (*I*) Stiffness of the bulge and HG in young and old back skin was measured ex vivo by AFM. (*J*) Quantification of stiffness (Young’s modulus) of bulge and HG in first telogen (P23), young (P54) and old back skin (26 mo old). Five hair follicles from two young animals and old animals were measured. For each area, 64 points were measured. For young hair follicles, Bu mean = 2240.1 ± 1251.1 Pa, HG mean = 824.2 ± 368.0 Pa. For old hair follicles, Bu mean = 2620.9 ± 1,868.0 Pa, HG mean = 1207.2 ± 625.2 Pa. *P* values were determined by the Mann–Whitney *U* test. Number following the ± sign shows SD. (*K*) Cortical F-actin signals were reduced, and cortical cytoskeletons were rearranged during the activation of hair growth. For quantification, mean and SD were shown from over 10 line-quantification values (*Right*). (*L*) pMLC2 signals were reduced in HG cells in early anagen, compared to telogen. Scale bar, 10 μm in A, C, E, G, I, K, and L. *P* values in F and K were determined by Student’s *t*-test. In *C* and *E*, the red line indicates the height of the hair germ (distance from the bottom of the bulge to the bottom of the hair germ) from the starting point. The yellow solid line indicates the width of the hair germ from the starting point. The yellow dash line indicates the position of the bottom of the hair germ from the starting point.

To determine cell dynamics within the HF-SC compartment, including both the bulge and HG (13, 14), during quiescence and activation, we performed noninvasive intravital imaging (15, 16). We monitored HF cells labeled with H2b-GFP (*Krt14-H2b-GFP* mice) beginning from late catagen (~postnatal day 19, P19) to early anagen (~P27). The size and position of individual HF-SCs in the bulge remained largely constant over the period. However, the size and shape of HG, which typically consists of ~10 to 20 stem and progenitor cells, underwent substantial (~35%) changes during the same period without any cell proliferation or death (Fig. 1*A* and *SI Appendix*, Fig. S1 *A* and *B*). To ensure the accuracy of H2b-GFP nuclear signals in measuring HG volume, we used *Krt14-H2b-GFP/Rosa-mTmG* to mark the nuclei and cell membrane, respectively. Double labeling revealed that stem and progenitor cells in the bulge and HG of HFs comprised mainly the nuclei with minimal cytoplasm. Therefore, the HG size quantified by H2b-GFP or membrane-localized tdTomato was similar for telogen and early anagen HGs (*SI Appendix*, Fig. S1 *C* and *D*). To minimize phototoxicity, we used H2b-GFP in subsequent studies since it allowed us to distinguish individual cells and cell division events at very low laser power. As shown in Fig. 1*A*, the HG contained the same number of cells but changed the volume, unlike the bulge, which remained largely unchanged from P19 (late catagen) to P24 (late telogen). For each HG, we designated the largest volume during observation as 100%. The smallest size reached ~54.56% of the largest volume (*SI Appendix*, Fig. S1*B*), usually during the middle of telogen. When the HFs transitioned toward anagen regeneration and cell cycle reentry, the size of HG increased (Fig. 1*A* and *SI Appendix*, Fig. S1*B*), with the average size of HG cells ranging from 454 μm^3^ to 735 μm^3^ (*SI Appendix*, Fig. S1*B*).

To determine the temporal dynamics of cell size changes in HF-SCs and HGs, we next performed intravital time-lapse imaging to quantify the relative size of telogen HFs. We recorded 31 HFs from nine animals every 6 min over 4 to 6 h, resulting in a total of 1,560 time points during telogen. Our results show that while the bulge HF-SCs remained largely unchanged, the HGs were dynamic. We designated the largest volume during observation as 100% and calculated the relative volume at each time point for comparison between HGs. We observed three types of HG activities—2 out of 31 (6.5%) continuously enlarged, 25 out of 31 (80.6%) dynamically enlarged and contracted, and 4 out of 31 (12.9%) contracted continuously (Fig. 1*B* and *SI Appendix*, Fig. S2*A*). For instance, one HG continuously contracted and reduced the volume by ~25% over 4 h during the first telogen at P22 (Fig. 1*C* and Movie S1). In contrast, the width and length of the bulge of the same HF remained constant (*SI Appendix*, Fig. S2*B*), and the relative position of one randomly selected cell to its two neighbors within the bulge remained unchanged (*SI Appendix*, Fig. S2*C*). Another HG enlarged and contracted intermittently in a 6-h span (Movie S2). The substantial contraction of HG during telogen was further confirmed when we visualized the adherens junction (AJ) on the cell membrane using an *E-cad-CFP* knock-in mouse (*SI Appendix*, Fig. S2*D* and Movie S3).

We next examined the dynamics of HG cells during the telogen-to-anagen transition when a new round of hair growth and cell cycle reentry were initiated. In early anagen, cell division was expected to increase the number of HG cells to fuel hair growth. Indeed, we observed cell division events and used them to mark the beginning of anagen (*SI Appendix*, Fig. S2 *E* and *F*). Infrequent cell divisions were observed in early anagen, such that there was no more than one cell division in a single HG over the time span of 4 to 6 h. Interestingly, cell division did not lead to significant increases in HG size (*SI Appendix*, Fig. S2*F*), consistent with experimental observations that cell division resulting from a larger cell gives rise to two smaller cells (12, 17). Overall, we found that HGs contracted less during early anagen than during telogen. Specifically, in 16 HFs from 5 mice, 25% of HGs exhibited continuous enlargement (Movie S4), while only 18.8% showed substantial (>10%) contraction (Fig. 1 *D* and *E* and *SI Appendix*, Fig. S2*G*). Similar to the telogen bulge, the bulge HF-SCs during the transition still remained largely unchanged (*SI Appendix*, Fig. S2 *H* and *I*). Quantification of the relative size of all HGs across 2,215 imaging time points (1,560 time points in telogen and 655 time points in anagen) revealed significantly reduced HG contraction in early anagen (9.77% of all HGs were smaller than 90% of the largest volume) compared to telogen (33.85% of all HGs were smaller than 90% of the largest volume) (Fig. 1*F*). Collectively, these intravital imaging data reveal that HG progenitor regions have different cell size dynamics in vivo compared to quiescent bulge HF-SCs. The quiescent bulge resists cell size changes, whereas HGs constantly change cell sizes during both quiescence and activation.

Cell size homeostasis has been linked to cell growth and global protein degradation in previous studies (12, 18–20). However, the differential size change observed in quiescent HF-SC and HG cells suggested a more dynamic mechanism modulating the cell size. We hypothesized that the spatially confined deformation patterns of the bulge and HG could be attributed to different mechanical properties, such as mechanical forces and resistance to the forces. We first used atomic force microscopy (AFM) to measure the stiffness of the bulge and HG in the first adult telogen and anagen ex vivo (*SI Appendix*, Fig. S3 *A*–*C*). Surprisingly, the bulge was 60% stiffer than HGs in the first telogen (1,196 Pa vs. 747 Pa) despite their spatial proximity. The stiffness of both bulge and HGs was reduced to 798 Pa and 375 Pa, respectively, during the first anagen when HF-SCs and HGs were activated to fuel hair growth (Fig. 1 *G* and *H*). Because the first telogen is very short, typically lasting 3 to 5 d, we further measured the stiffness of the bulge and HG during the prolonged second telogen and the extended telogen in old mice. Both bulge and HGs became progressively stiffer such that the stiffness of the bulge and HG was 2,240 Pa and 824 Pa, respectively, in P54 samples and 2,620 Pa and 1,207 Pa, respectively, in 26-mo samples (Fig. 1 *I* and *J*). The mechanical architecture of the HF-SC compartment, where HF-SCs are stiffer than HG progenitors, differs from that of the epidermis, where differentiated cell layers were >4 times stiffer than the basal SC layer (7).

Next, we examined internal force generation by the bulge and HG cells. Previous studies demonstrated that the actomyosin network plays important roles in pulsed cellular activities during tissue morphogenesis, including apical constriction during gastrulation of *Drosophila melanogaster* and *Caenorhabditis elegans* (21, 22), dorsal closure (23) and germband extension (24) of *Drosophila*, and compaction of mouse embryos (25). We quantified filamentous actin (F-actin) in both bulge HF-SCs and HG cells during telogen and the telogen-to-anagen transition. We used Ki67 staining to mark the onset of anagen as an indicator of an active cell cycle. In telogen, HF-SCs located in the bulge had very strong F-actin signals with robust stress fibers across the cells. During early anagen, actin organization transitioned from stress fibers to more cortical organization (Fig. 1*K*). In contrast to bulge HF-SCs, HG cells exhibited weaker F-actin signals characterized by cortical organization. However, F-actin bundles in HG cells were also rearranged during the activation of anagen hair growth. In telogen, cortical F-actin had stronger signals and was evenly distributed underneath the plasma membrane. At the onset of anagen (anagen I), as Ki67-positive (Ki67+) cells appeared in the HG, F-actin signals became weaker and more diffuse. As the hair cycle progressed to anagen II and more HG cells became Ki67+, HG cells exhibited more diffuse and weaker cortical F-actin signals than their telogen counterparts (Fig. 1*K*). Consistent with these patterns of F-actin, phosphorylated myosin light chain 2 (pMLC2), a well-established effector for actomyosin contractility, was strong in both the bulge and HG during telogen, but its intensity was reduced in HGs during early anagen (Fig. 1*L*).

We next investigated how actomyosin contractility is translated into biochemical signals that activate cell cycle reentry in HG cells. To this end, we examined the correlation between F-actin signal strength and nuclear YAP, a mechanosensitive indicator and signal transducer (5, 26, 27), during telogen and early anagen. YAP signals were weak and diffuse in the cytoplasm during telogen (*SI Appendix*, Fig. S4*A*). However, YAP accumulated in the nuclei of a few HG cells when transitioning into early anagen (*SI Appendix*, Fig. S4*B*), and the nuclear accumulation spread to nearly all HG cells by late anagen II (*SI Appendix*, Fig. S4*C*). Coquantification of cortical F-actin and nuclear YAP signals within individual cells revealed an inverse correlation between cortical actomyosin cytoskeleton and nuclear accumulation of YAP (*SI Appendix*, Fig. S4*D*). A randomized test with 1,000 resamplings indicated that the inverse correlation was highly significant (*P* < 0.001) (*Materials and Methods*). Furthermore, a combined F-actin and nuclear YAP score accurately distinguished telogen and anagen states in a mathematical model (*SI Appendix*, Fig. S4 *E* and *F*). Together, these data indicate that the weakened actomyosin contractility, correlated with nuclear YAP accumulation, initiates HG proliferation to fuel hair growth at the onset of anagen.

### miR-205 Induction Promotes Hair Regeneration.

**Fig. 2.**
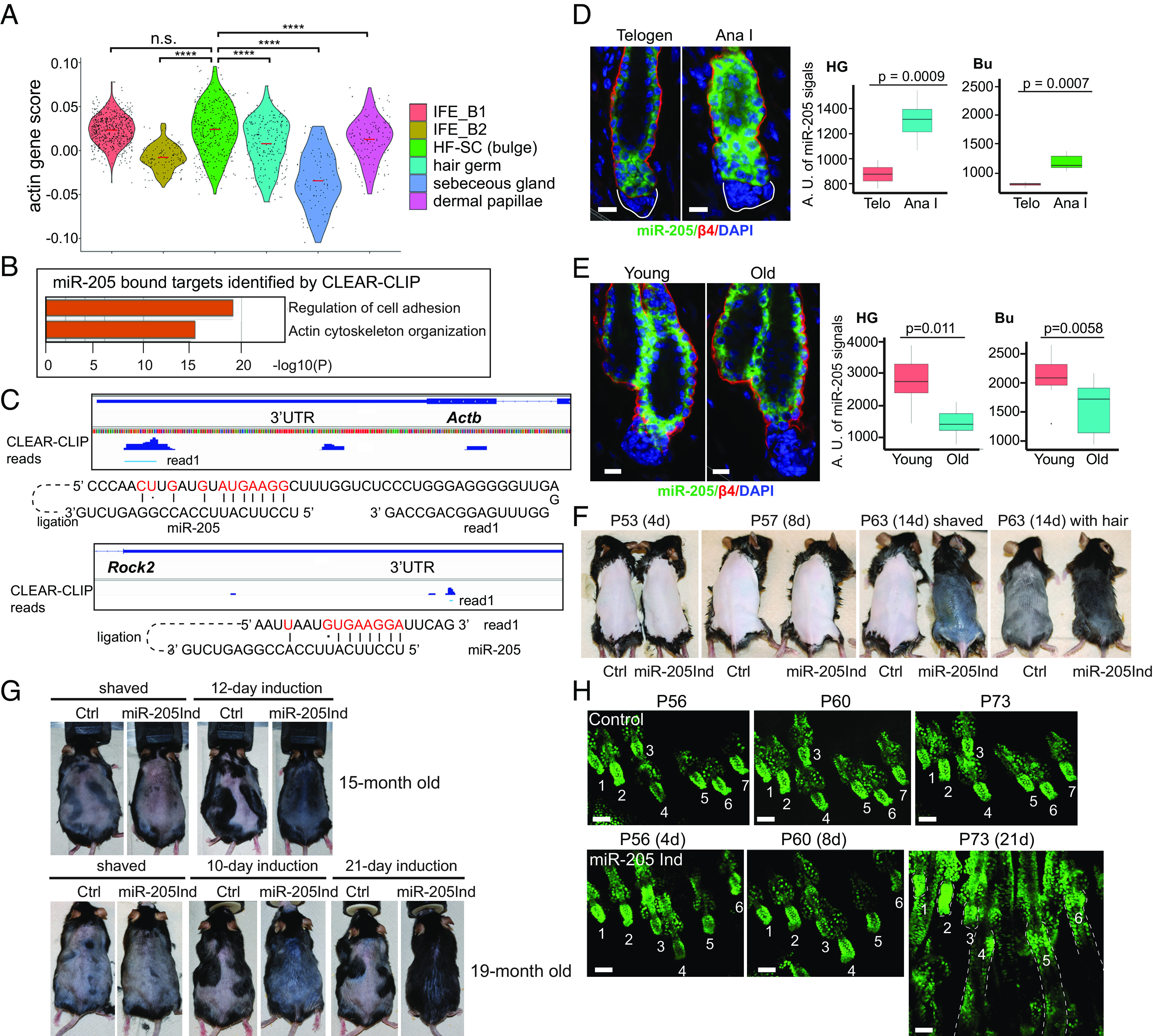
*miR-205* induction promotes hair regeneration in young and old mice. (*A*) Epithelial and dermal cell populations from telogen skin had different actin gene expression scores. ****P < 0.001, n.s., not significant. IFE_B: interfollicular epidermis basal cell; HF-SC: hair follicle stem cells. (*B*) *miR-205* bound targets were enriched in components and regulators of the actin cytoskeleton and cell adhesion, determined by CLEAR-CLIP. (*C*) Binding of *miR-205* to target sites in the 3′UTR of *Actb* and *ROCK2* was captured by CLEAR-CLIP. Base-pairing between *miR-205* and targeted sites, marked by red font, is shown. (*D*) In the bulge and HG, the expression of *miR-205* was elevated by ~50% from telogen to the initiation of anagen in the bulge and HG, detected by fluorescent in situ hybridization. Quantification for HG and Bu is shown in the right panel. For quantification, 4 telogen and 6 anagen hair follicles were used. (*E*) The expression of *miR-205* was decreased by ~50% between young and old telogen HG and ~ 20% between young and old bulge, detected by fluorescence in situ hybridization. Quantification for HG and bulge is shown in the *Right* panel. Six young hair germs and 10 old hair germs as well as 14 young and 13 old bulges were used for quantification. (*F*) *miR-205* promoted hair growth in second telogen, starting induction at P49. Of note, eight pairs, three pairs, and seven pairs of animals were used for 4-d, 8-d, and 14-d induction phenotypical analysis, respectively. (*G*) *miR-205* promoted hair regeneration in old mice (15-mo and 19-mo-old). Four pairs and three pairs of animals were used for 15-mo and 19-mo induction phenotypical analysis, respectively. (*H*) *miR-205* promoted ear hair follicle regeneration in young mice, observed with a multiphoton microscope. (Scale bar, 10 μm in *D* and *E*, 100 μm in *H*). *P* values were determined by Student’s *t*-test.

We next examined whether the differences in actin cytoskeleton and contractile behaviors observed in bulge HF-SCs and HG cells were associated with differential gene expression. We calculated an actin-related gene expression score (*Materials and Methods*) for epithelial and dermal cell populations of the skin by analyzing single-cell RNA-seq datasets from telogen skin (16). HF-SCs located in the bulge had the highest actin score, correlating with the strongest F-actin bundle formation and high pMLC2 levels. HG progenitors had lower actin scores than that of HF-SCs (Fig. 2*A*), consistent with the weaker actomyosin network in the HG. Interestingly, two basal populations of the interfollicular epidermis (IFE), commonly identified in scRNA-seq dataset (28), had different actin scores, indicating that different actin cytoskeleton contributes to epidermal heterogeneity. Sebaceous glands had the lowest actin score among major epithelial populations.

We next asked whether we can modulate actomyosin cytoskeleton and force generation by perturbing the expression of actin regulators. We turned to miRNAs, which are known to posttranscriptionally down-regulate many genes on a relatively minor scale and in a reversible manner (29, 30). We performed genome-wide capture of miRNA targets together with their associated miRNAs, which detected individual miRNA:mRNA interactions through a direct ligation of miRNA and targeted mRNA site when they were present in the same miRNA-induced silencing complex (31). By analyzing all miRNA–mRNA targeting pairs, we found that *miR-205*, one of the most highly expressed miRNAs in epidermal stem and progenitor cells (32), targets many components and regulators of actomyosin cytoskeleton and cell adhesion. Genes involved in force generation by myosin II activation, such as kinase *Rock2*, and F-actin polymerization, such as *Actb* and *Actn4,* as well as AJ components and force transducers, such as *Ctnna1* (encoding α-catenin), were broadly targeted by this miRNA (Fig. 2 *B* and *C* and *SI Appendix*, Fig. S5 *A* and *B*). Notably, *miR-205* also directly bound to *Piezo1*, a mechanosensitive calcium channel gene, at an evolutionarily conserved site in the 3′UTR (*SI Appendix*, Fig. S5*B*).

We next examined the expression and regulation of *miR-205.* The expression of *miR-205*, determined by quantitative fluorescent in situ hybridization, was elevated by ~50% from telogen to the initiation of anagen in the bulge and HG (Fig. 2*D*). Furthermore, *miR-205* was down-regulated both in the bulge and HG in old mice (Fig. 2*E*). *miR-205* is transcriptionally regulated by ΔNp63 through three enhancers (*SI Appendix*, Fig. S6*A*), the master transcription factor for specifying the epithelial fate of the skin (33). Interestingly, ΔNp63 expression was low and sparse in both HF-SCs and HGs in telogen, compared to their anagen counterparts, while ΔNp63 expression was generally strong and uniform in the IFE basal cells (*SI Appendix*, Fig. S6 *B*–*D*). In old mice, ΔNp63 expression was significantly reduced in both HF-SCs and HGs, compared to young mice (*SI Appendix*, Fig. S6 *E*–*G*). Thus, decreased *miR-205* expression was correlated with reduced hair regeneration in old mice.

We next tested whether *miR-205* induction controls bulge HF-SCs and HG cells and modulates hair regeneration. We first induced *miR-205* during early telogen of the second adult hair cycle at P42 (*SI Appendix*, Fig. S7 *A* and *B*). Usually, the second telogen lasts more than 30 d from P42 to P70 (13). Here, we found that by 5 d after the initial induction (P47), the hair cycle of the dorsal skin transitioned to anagen II-III. By 7 d postinduction (P49), the hair cycle reached anagen IV accompanied by the appearance of darkened skin, caused by terminal differentiation of the melanocytes (*SI Appendix*, Fig. S7 *C* and *D*). The rapid telogen-to-anagen transition caused by *miR-205* was unusual. In comparison, in mice with genetic deletion of *Foxc1*, which is a key transcription factor (TF) governing HF-SC quiescence through the control of BMP and FGF signaling, it takes ~14 d to reach anagen III (34, 35). To further examine the potency of *miR-205* induction on promoting hair growth, we induced *miR-205* at P49, when the dorsal HFs were uniformly in refractory telogen with high BMP signaling (36). By P53, 4 d after the initial *miR-205* induction, both control and induced HFs still appeared to rest in telogen. By P57, 8 d after the initial induction, most dorsal HFs entered anagen III when HF downgrowth was readily observed. By P63 (14 d of induction), the dorsal hair coat was regenerated (Fig. 2*F* and *SI Appendix*, Fig. S7*E*). During aging, the duration of quiescent telogen gradually increases, and it often lasts >100 d in mice more than 1 y old (37). To test the efficacy of *miR-205* induction in old animals, we examined 15-mo-old and 19-mo-old mice. In all cases examined, *miR-205* induction resulted in robust and uniform hair growth typically within 14 d, judging by the appearance of darkened skin color and hair coat regeneration (Fig. 2*G*).

To track the progression of hair growth of individual HFs in live animals, we used 2-photon microscopy to monitor the same HFs (ear) in control and *miR-205-*induced animals. Although HFs in the ear were more refractory to the initiation of hair growth (38), *miR-205* induction still initiated anagen hair growth, judging by the HG morphology, within 14 d in young (~P52), middle-aged (~12-mo), or older (~15-mo) mice (Fig. 2*H* and *SI Appendix*, Fig. S7*F*). Thus, *miR-205* induction potently promotes hair regeneration regardless of the age or the inhibitory microenvironment.

### miR-205 Inhibits Actomyosin Contractility and Force Generation.

**Fig. 3.**
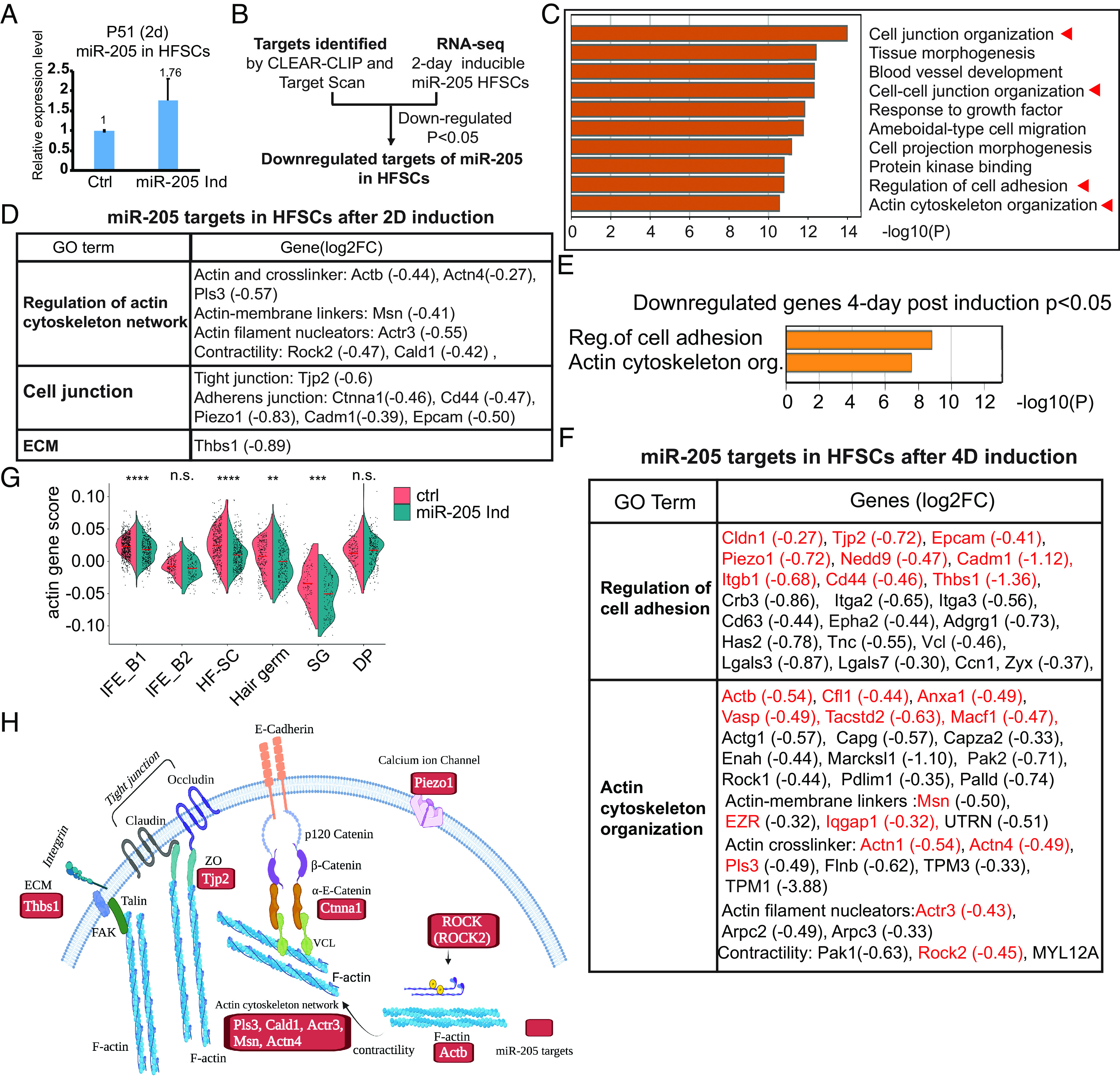
*miR-205* targets actomyosin network and cell adhesion genes. (*A*) The *miR-205* level was increased by ~1.76 fold 2 d after induction in FACS-purified HF-SCs. (*B*) Workflow of *miR-205* target identification by combining CLEAR-CLIP capture and TargetScan prediction (for target site conservation) and bulk RNA-seq in HF-SCs. (*C*) *miR-205* targets, enriched in the gene categories of cell junction and the actin cytoskeleton, are the most highly enriched gene groups among down-regulated genes in HF-SCs after 2-d induction. (*D*) *miR-205* targets, enriched in the gene categories of cell junction and the actin cytoskeleton, were broadly down-regulated in HF-SCs after 2-d induction. (*E*) Genes associated with the regulation of cell adhesion and actin cytoskeleton organization are the most highly down-regulated genes in *miR-205*-induced HF-SCs 4 d after induction. (*F*) Down-regulated genes in the categories of cell adhesion and actin cytoskeleton organization in *miR-205*-induced HF-SCs 4 d after induction. *miR-205* targeted genes are highlighted in red color. (*G*) *miR-205* reduced the actin score in the actin^hi^ basal epidermis, HF-SCs, HGs, and SGs but not in DP. (*H*) Illustration of widespread regulation of the actomyosin network, multiple cell adhesion machinery, including the adherens junction, tight junction, and integrin, as well as *Piezo-1* by *miR-205*. Experimentally identified *miR-205* targets are highlighted in red color.

To determine the underlying mechanism of *miR-205*-mediated hair regeneration, we performed bulk RNA-seq with FACS-purified HF-SCs 2 d after the induction during the refractory telogen before any signs of cell proliferation and anagen reentry. While *miR-205* was only mildly induced by ~1.8-fold (Fig. 3*A*), it caused widespread downregulation of targeted genes involved in actin cytoskeleton, cell adhesion, and junctions (Fig. 3 *B* and *C*). This result confirmed that *miR-205* down-regulated genes involved in the regulation of actin cytoskeleton upon the induction in HF-SCs in vivo, including *Actb, Actn1, Actn4, Ctnna1, Rock2,* and *Piezo1,* identified by the direct ligation of *miR-205* and targeted sites (Fig. 3*D* and Dataset S1). We next examined the impact of *miR-205* on the transcriptome of epithelial cell populations by performing scRNA-seq 4 d after the induction when HFs transition from telogen to anagen. Unchanged UMAP cell clustering between control and *miR-205-*induced samples indicated that *miR-205* induction did not globally perturb cell fate (*SI Appendix*, Fig. S8 *A*–*D*). Consistent with the bulk RNA-seq data, *miR-205* targets, including the ones involved in myosin II activation, F-actin bundle formation, and cell-junction organization, were broadly down-regulated in both HF-SCs and HGs (Fig. 3 *E* and *F* and Dataset S2). Interestingly, the actin score, a composite index of actin-related gene expression, was significantly down-regulated in the actin^hi^ IFE, HF-SCs, HGs, and SGs but not in the actin^lo^ IFE and dermal papillae in the *miR-205-*induced sample (Fig. 3*G*), confirming the broad impact of *miR-205* induction on actin cytoskeleton in the epithelial cells. In HF-SC and HG cell clusters, the most highly enriched gene groups among the down-regulated *miR-205* targets were actin cytoskeleton and cell adhesion (Fig. 3 *F* and *H*). Furthermore, genes associated with response to FGF signaling, regulation of mitotic cell cycle, and active WNT signaling were enriched among up-regulated genes in *miR-205-*induced HGs (*SI Appendix*, Fig. S8 *E* and *F*), consistent with the gene signatures detected during normal telogen-to-anagen transition (13). Together, these data indicate that *miR-205* suppresses genes associated with the actomyosin cytoskeleton and promotes anagen activation.

**Fig. 4.**
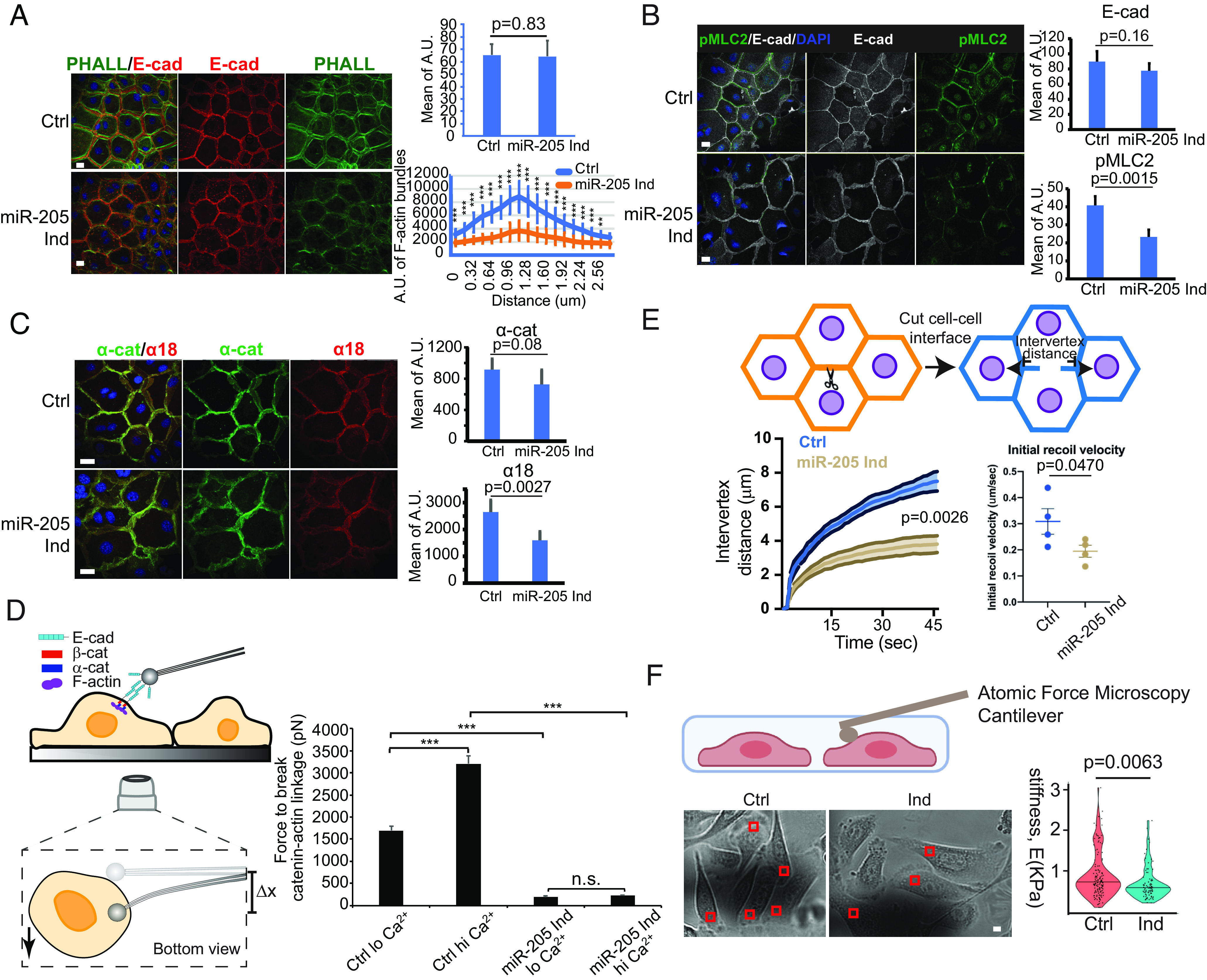
*miR-205* inhibits actomyosin contractility and force generation and reduces cell stiffness. (*A*) *miR-205* reduced cortical F-actin bundle formation but not E-cadherin membrane localization. For E-cadherin and PHALL data, 9 control and 15 *miR-205* induction images were used for quantification (*Right*). F-actin bundle data (*Lower Right*) were mean and SD from over 10 line-quantification values. (Scale bar, 20 μm). **P < 0.01, ***P < 0.001, ****P < 0.0001 determined by Student’s *t*-test. (*B*) *miR-205* reduced pMLC2 levels in keratinocytes. Three control and 6 *miR-205* induction images were used for quantification (*Right*). *P* values were determined by Student’s *t*-test. (Scale bar, 20 μm). (*C*) *miR-205* reduced actomyosin forces on α-catenin (reflected by α18 signals). Seven control and 4 *miR-205*-induced images were used for quantification (*Right*). *P* values were determined by Student’s *t*-test. (Scale bar, 10 μm.) (*D*) *miR-205* reduced the mechanical forces generated by the AJs and the underlying actomyosin networks in keratinocytes with and without calcium-induced differentiation. ***P < 0.001 determined by Student’s *t*-test. (*E*) *miR-205* decreased cell membrane tension. For the initial recoil velocity assay, the *P* value was determined by the paired *t*-test. For intervertex distance measurement, the *P* value was determined by two-way ANOVA. Both graphs showed the average of four independent experiments with the error bars as SEM. (*F*) *miR-205* reduced stiffness of culture cells. A total of 20 control cells from 2 plates and 23 *miR-205* inducible cells from 2 plates were used for quantification. Control cells, mean = 910.6 ± 542.8 Pa; cells after *miR-205* induction, mean = 734.4 ± 395.4691Pa. *P* values were determined by the Mann–Whitney *U* test. Number following the ± sign shows SD (Scale bar, 10 μm).

We next examined how elevated *miR-205* expression regulates actomyosin contractility and mechanical property of the cells by using primary keratinocyte isolated from the inducible model. Consistent with in vivo induction, *miR-205* levels were induced by ~75% 24-h postinduction (*SI Appendix*, Fig. S9*A*). Importantly, induction of *miR-205* reduced cortical F-actin bundle formation without changing the levels of E-Cad, which is not a target (Fig. 4*A*). Phosphorylated myosin light chain 2 (pMLC2), an indicator for actomyosin contractility and regulated by ROCK2 (39–41), was reduced by ~40% in *miR-205-*induced keratinocytes (Fig. 4*B*). Furthermore, whereas the levels of α-catenin (*Ctnna1)*, which is a target of *miR-205,* were mildly reduced (~20%) by *miR-205* as expected, α18 signals were reduced more strongly by ~40% (Fig. 4*C*), reflecting the reduction of mechanical forces on a-catenin.

To investigate the effect of *miR-205*-mediated regulation on mechanical forces generated by the AJ and the actomyosin cytoskeleton, we conducted an E-cad microbead displacement experiment (6) to quantify the forces required to break the interaction between AJs and the underlying actomyosin network. In low Ca^2+^–cultured keratinocytes, control cells required ~1,700 pN of force to break the AJ–actin linkage, whereas the breaking forces were elevated to ~3,300 pN in high Ca^2+^–cultured, differentiated keratinocytes due to the increased bond between the AJ and the actin cytoskeleton upon Ca^2+^ stimulation (Fig. 4*D*). Strikingly, *miR-205* induction reduced the mechanical forces required to break the AJ–actin linkage by ~10-fold, regardless of the Ca^2+^ concentration in the media (Fig. 4*D*). Notably, genetic deletion of *VCL*, a key connector of AJs to the actin cytoskeleton, reduced the mechanical forces only by ~two fold in the same assay (6). In mice, genetic deletion of *VCL* significantly reduced F-actin and α18 signals and caused widespread cell cycle reentry of both HF-SCs and HGs (*SI Appendix*, Fig. S9 *B* and *C*), directly linking AJ-mediated force generation to the control of cell cycle reentry. Collectively, these results demonstrate that *miR-205* substantially reduces the AJ–actin force generation by cotargeting myosin II activation, F-actin bundle formation, and AJ components.

Next, we asked whether *miR-205* induction also perturbed the tension on the plasma membrane because actomyosin contractility could affect mechanical property on the cell surface. Using a laser ablation and recoil assay (42, 43), we observed that the intervertex distance decreased by ~50% within 45 s after the laser ablation, and the initial recoil velocity decreased by ~33% (Fig. 4*E*). To further corroborate these findings, we used AFM to measure the stiffness of the cell surface upon *miR-205* induction. Interestingly, the stiffness of the cell surface was reduced from 910 Pa in control cells to 734 Pa in *miR-205*-induced cells. Together, these data demonstrate that *miR-205* induction reduces mechanical forces generated by the AJs and actomyosin contractility at the cortex as well as decreases the stiffness and membrane tension of the cell surface.

### Mechanism of miR-205-Mediated Hair Regeneration.

**Fig. 5.**
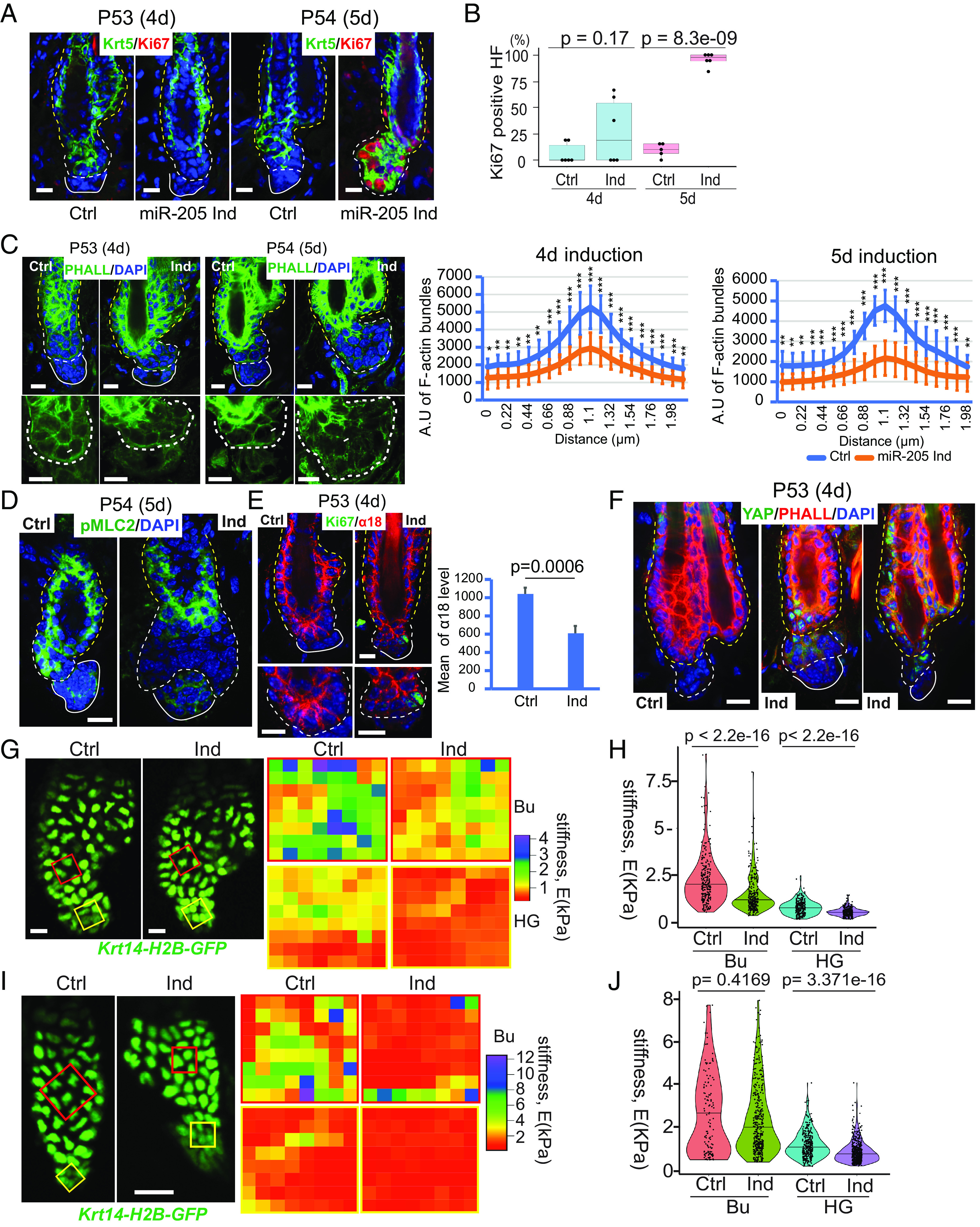
*miR-205* modulates mechanical stiffness and actomyosin contractility of HF-SC compartment. (*A*) *miR-205* promoted Ki67 accumulation 4 to 5 d after induction. (*B*) The percentages of Ki67+ HFs were quantified after 4-d and 5-d induction. A total of 71 control HFs and 57 *miR-205*-induced HFs from six pairs of animals for 4-d induction, 61 control HFs from five animals, and 145 *miR-205*-induced HFs from six animals for 5-d induction, respectively, were used. *P* values were determined by Student’s *t*-test. (*C*) *miR-205* reduced cortical F-actin levels and modulated the actomyosin network after 4-d and 5-d induction. F-actin bundle data (*Right*) were mean and SD from over 10 line-quantification values. (*D*) *miR-205* reduced pMLC2 levels in HG cells after 5-d induction. (*E*) *miR-205* reduced actomyosin forces on a-catenin (reflected by α18 signals). Seven control and 4 *miR-205*-induced images were used for quantification (*Right*). *P* values were determined by Student’s *t*-test. (*F*) *miR-205* remodeled the actomyosin cytoskeleton and resulted in nuclear YAP accumulation in both HF-SCs and HG cells after 4-d induction. (*G*) *miR-205* reduced the stiffness of both the bulge and HG compartments after 5-d induction in young mice, determined by AFM ex vivo. (*H*) The stiffness of both the bulge and HG compartments was reduced in *miR-205*-induced young hair follicles. Bu in control skin, mean = 2240.1 ± 1251.1 Pa; Bu in miR-205 samples, mean = 2030.8 ± 1570.6 Pa. HG in control samples, mean = 824.2 ± 368.0 Pa; HG in miR-205 skin, mean = 505.7 ± 244.6 Pa. (*I*) *miR-205* reduced the stiffness of both the bulge and HG compartments after 7-d induction in old mice, determined by AFM ex vivo. (*J*) The stiffness of both the bulge and HG compartments was reduced in *miR-205*-induced hair follicles in old mice. Four control and 5 *miR-205* inducible hair follicles from two pairs of animals were used for quantification. Bu in control skin, mean = 2620.9 ± 1,868.0 Pa; Bu in miR-205 samples, mean = 2335.6 ± 1,603.0 Pa. HG in control samples, mean = 1207.2 ± 625.2 Pa; HG in miR-205 skin, mean = 886.9 ± 501.9 Pa. *P* values were determined by the Mann–Whitney *U* test. Number following the ± sign shows SD (Scale bar, 10 μm in *A*, *C*, *E*, *F*, *G*, and *I*, 15 μm in *D*).

Having established the role of *miR-205* in modulating actomyosin contractility and membrane tension in vitro, we examined cellular responses to *miR-205* induction in vivo. The appearance of Ki67+ cells in the HG marks the onset of anagen initiation. In *miR-205-*induced skin, Ki67+ HG cells began to emerge sporadically 4 d after the initial induction (P49-P53), and ~20% of HFs contained Ki67+ HG cells. By 5 d after the induction, however, nearly 100% of HFs contained Ki67+ HG cells (Fig. 5 *A* and *B*). These data were consistent with our transcriptome analysis for the effect of *miR-205* induction. Indeed, cortical F-actin bundles were reduced in HG cells 4 d after the induction (Fig. 5 *C*, *Left*). By 5 d after the induction, cortical F-actin signals were further weakened and became diffuse with clear signs of cell number increase in the HG (Fig. 5 *C*, *Right*), reflecting cell cycle reentry. Consistent with these morphological changes in actomyosin networks, mechanical properties of HG cells also changed, indicated by reduced pMLC2 signals in the HG (Fig. 5*D*) and reduced (~40%) and punctate α18 signals that reflect weakened actomyosin forces on a-catenin (Fig. 5*E*). The reduced α18 signals in *miR-205*-induced HGs were similar to those of the normal telogen-to-anagen transition, observed in control mice (*SI Appendix*, Fig. S9*D*). Accompanying these changes, YAP protein began to accumulate in the nuclei of not only HG cells but also bulge HF-SCs 4 d after *miR-205* induction (Fig. 5*F*). Furthermore, modeling of cortical F-actin and nuclear YAP signals obtained from *miR-205-*induced samples revealed that *miR-205* induction promoted the transition of HG cells from the quiescent telogen state to the activated anagen state (*SI Appendix*, Fig. S9 *E* and *F*). In addition to the changes in actin cytoskeleton, *miR-205* induction was also correlated with reduced stiffness of both the bulge and HG compartments in both young and old samples, determined by AFM ex vivo (Fig. 5 *G*–*J*). In P54 samples, bulge stiffness was reduced from 2,240 Pa to 2,030 Pa, and HG stiffness was reduced from 824 Pa to 505 Pa (Fig. 5 *G* and *H*). Interestingly, the stiffness of the bulge (2,620 Pa) and HG (1,207 Pa) in 21- to 22-mo-old mice was reduced to the levels usually found in young mice (bulge, 2,335 Pa; HG, 887 Pa) upon *miR-205* induction (Fig. 5 *I* and *J*).

**Fig. 6.**
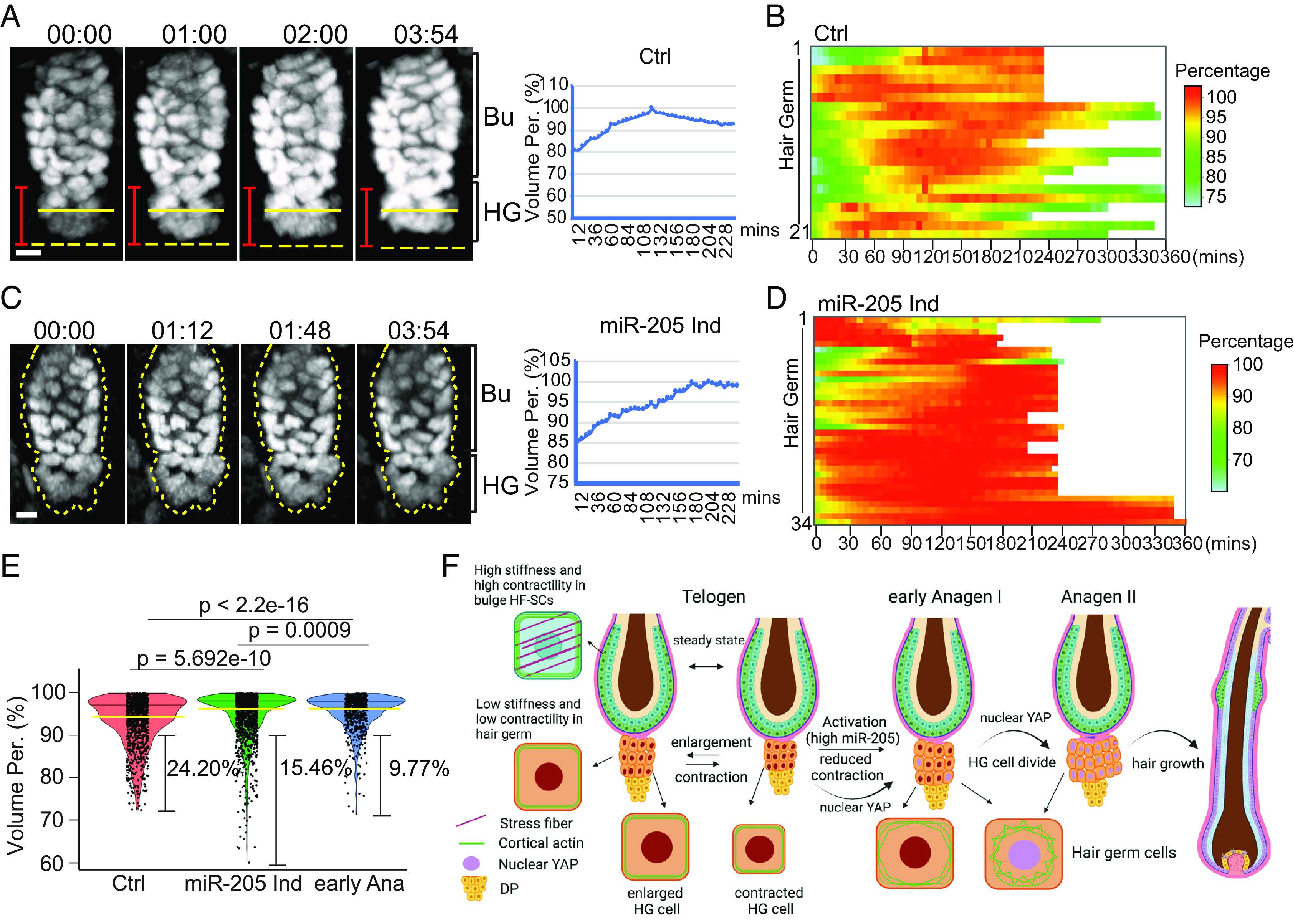
Interplay between tissue stiffness, actomyosin contractility, and cell size control during the transition between quiescence and activation of hair regeneration. (*A*) An example of HG undergoing enlargement and contraction (*Left*) with quantification (*Right*) over 4 h in second telogen. (*B*) HGs showed the behaviors of contraction and enlargement in second telogen. A total of 21 hair follicles in second telogen from seven control animals were quantified. (*C*) An example of HG undergoing continuous enlargement (*Left*) with quantification (*Right*) in *miR-205*-induced sample over 4 h. (*D*) *miR-205* reduced HG contraction; 34 hair follicles from 8 *miR-205-*induced animals were recorded. (*E*) miR-205-induced HGs reduced contraction, mimicking the behavior observed in early anagen of control mice. The yellow lines represent the median values, and the black lines represent the third quarter of all time points in the violin plot. (*F*) Schematic illustration of the interplay between tissue stiffness, actomyosin contractility, nuclear YAP accumulation, and cell size dynamics during the transition between quiescence and activation of hair regeneration. (Scale bar, 10 μm in *A* and *C*). *P* values were determined by Student’s *t*-test.

Because *miR-205* reduced actomyosin contractility in the bulge and HG, we next asked whether *miR-205* changes contractile behaviors and cell size dynamics of HGs in live animals. During the second, prolonged telogen, time-lapse live imaging revealed the same enlargement and contraction activities of quiescent HGs. In 21 HFs from 7 mice, 19 (90.5%) HGs exhibited dynamic enlargement and contraction with a size change of ~20% and 2 (9.5%) HGs only underwent enlargement during the 4 to 6-h imaging window (Fig. 6 *A* and *B*). These cell behaviors were reminiscent of the behavior of HGs in the first telogen, during which 93.5% of HGs (29 out of 31 HFs) exhibited substantial (>10%) contraction (Fig. 1*B*). In contrast, only 9 (26.5%) HGs showed substantial (>10%) contraction, whereas the remaining 73.5% of HGs did not contract or mildly increased their size in 34 HFs from 8 induced mice (Fig. 6 *C* and *D* and Movie S5). Thus, *miR-205* induction significantly reduced HG contraction, defined by the percentage of HGs whose size was less than 90% of the largest volume during observation (Fig. 6*E*). Collectively, these results demonstrate that *miR-205* induction reduces cell size contraction and promotes cell cycle reentry through reduced actomyosin contractility in the bulge and HG.

## Discussion

### Spatiotemporally Defined Mechanical Properties of HF-SC Niche.

In this study, we have demonstrated spatiotemporal compartmentalization of mechanical properties and their associated cell activities within the HF-SC niche in live animals (Fig. 6*F*). Bulge HF-SCs are stiff and exhibit strong actomyosin contractile forces, while HG progenitors are relatively soft and mechanically dynamic. We note that the measurement of stiffness in sliced HFs using the contact-based AFM method likely probed the composite that was in direct contact with the cantilever. To address this issue, we selected only HFs whose epithelial–dermal interface was exposed (*SI Appendix*, Fig. S3*A*) and measured 64 points in a 15-μm × 15-μm region for each HF. As a result, we obtained similar results for young and old HFs, as previously reported (8). Although induction of *miR-205* reduces actomyosin contractility in both the bulge and HG, only HG cells readily reenter the cell cycle and initiate HF growth. These results suggest that HG progenitor cells are more sensitive to the change of mechanical forces and rapidly mobilize to initiate HF regeneration, whereas HF-SCs are more insulated and protected by high actomyosin contractility of individual HF-SCs and the stiffer microenvironment. The spatial compartmentalization of stiff and quiescent HF-SCs and soft and primed HGs is different from the architecture of the epidermis, where the basal, proliferative SC layer is soft, and the suprabasal, differentiated layer is stiff (7). Interestingly, our single-cell transcriptomic analysis suggests that two basal SC populations have different actin scores (Fig. 2*A*). This raises a possibility that these two populations may also have distinct mechanical properties and respond differently to the changes of forces. For HF regeneration, previous studies have revealed a stepwise activation mechanism of bulge HF-SCs and HGs, controlled by signaling pathways such as FGF and SHH (13, 14). This study has characterized the mechanical heterogeneity of HF-SCs and HG progenitors and has demonstrated that mechanical properties modulate cellular activities.

### Widespread Targeting of Actomyosin Machinery by miR-205 Modulates Cell Mechanics.

Mechanistically, *miR-205* inhibits many genes involved in the regulation of the actomyosin network as well as *Piezo1*. Genome-wide detection of *miR-205* targeted mRNAs through direct ligation of *miR-205* and target sites allows unbiased identification of most targets and predicts the functional impact of this miRNA in the regulation of cell mechanics. This approach, in combination with both bulk and single-cell RNA-seq, globally assesses miR-205-mediated gene regulation and function in the bulge and HG cells. Although each mRNA is only mildly reduced upon *miR-205* induction, the collective impact on cell mechanics is substantial. Interestingly, *miR-205* is transcriptionally targeted by ΔNp63, whose expression together with *miR-205* is reduced in old HF-SCs and HGs particularly in telogen. ΔNp63 has been implicated in epithelial stem cell self-renewal, while *miR-205* modulates actomyosin contractility to promote cell cycle reentry. The concurrent decreases in their expression in old HF-SCs are consistent with reduced ability to self-renew and increased stiffness during aging (8, 44).

We note that *miR-205* has been previously shown to target negative regulators of PI3K pathways, including *Pten* and *Inppl1,* in cultured cells and during embryonic skin development (32, 45). However, this layer of regulation appears to be relatively minor in adult HF-SC and HG cells because we detected low expression of these targets and only minor changes of their expression upon *miR-205* induction. Furthermore, genetic deletion of *Pten* in adult HF-SCs fails to activate HF-SCs and HF regeneration in 4 weeks (46). This makes it unlikely that *miR-205-*mediated regulation of PI3K pathways is a major contributor for the observed phenotype. However, it is possible that the repression of other targets of *miR-205* plays a key role to promote cell cycle reentry and hair regeneration.

Functionally, whereas *Vinculin-*deleted HFs eventually degenerate due to permanent deletion of this cytoskeleton gene (6), induction of *miR-205* rapidly promotes hair regeneration in both young and old mice through mild and reversible downregulation of many genes. Furthermore, perturbation of cell mechanics by *miR-205* induction overrides the inhibitory BMP signaling and correctly activates FGF and WNT signaling for hair regeneration. The reversible gene expression regulation and potent effect as well as the possibility to deliver small RNA through nanoparticles subcutaneously (47) nominate *miR-205* as a candidate to stimulate adult hair regeneration by transiently modulating cell mechanics through fine-tuning gene expression.

Finally, cell size control and mechanics play fundamental roles in cell physiology and tissue homeostasis (1, 2, 12, 18, 48). This study has implicated dynamic changes in cell size and subsequent cell cycle reentry as a mechanism for sensing and responding to the changes of mechanical forces during homeostasis, wound repair, and aging.

## Materials and Methods

### Cell Lines.

Primary keratinocytes were isolated from mice between the ages of P0 and P4 as previously described, with some modifications (49). Mice were killed, back and belly skin was collected, and excess fat was scraped from the tissue. The skin was then placed dermal side down in 2× dispase for 30 to 60 min at 37 °C. The epidermis was subsequently separated from the dermis with forceps and placed into 0.05% trypsin–EDTA for 10 min at 37 °C. Trypsin was quenched with culture media, and cells were strained through a 40-µm filter and subsequently plated in E-low Ca^2+^ media onto dermal feeder cells.

### Animal Studies.

All mice breeding and operation procedures were approved by the institutional animal care and use committees at the University of Colorado Boulder (CO, USA) and at Northwestern University Feinberg School of Medicine (IL, USA) and in accordance with the guidelines and regulations for the care and use of laboratory animals.

The miR-205 induction mouse line was generated through standard transgenic injection of the linearized *pTRE2-miR-205* DNA into FVB mice. Founders were bred with mice harboring a keratin14 reverse tetracycline trans-activator (*Krt14*-rtTA, E. Fuchs, Rockefeller University) to produce mice with skin-specific doxycycline-inducible expression of the miR-205. Multiple lines were established and validated for the study. Doxycycline chow used in experiments was 625 mg/kg (Teklad rodent diet TD-7012). *E-cadherin-mCFP* (JAX # 016933), and *Rosa-mTmG* (JAX # 007676) mice were obtained from the Jackson laboratory. *Krt14*-*H2b*-*GFP* (E. Fuchs, Rockefeller University), *E-cadherin-mCFP*, and *Rosa-mTmG* mice were used for live animal imaging.

### Immunostaining, In Situ Hybridization, and Imaging.

For analysis of back-skin phenotypes, 10-μm OCT sections were fixed in 4% PFA for 10 min in phosphate-buffered saline (PBS) and washed three times for 5 min in 1× PBS at room temperature. The sections were blocked with 2.5% NGS and 2.5% NDS in PBS. Sections were incubated with primary antibody overnight at 4 °C (50). After incubation with primary antibodies, sections were washed three times in 1× PBS for 5 min and incubated for 1 h at room temperature with Alexa Fluor 594–, Alexa Fluor 488–, or Alexa Fluor 647–conjugated secondary antibodies (1:2,000; Invitrogen/Molecular Probes). Alexa Fluor™ Plus 555 Phalloidin (1:200; A30106; Invitrogen) or Alexa Fluor 488 conjugated phalloidin (1:50; A12379; Invitrogen) was used to stain F-actin. For pMLC2 staining, fluorescent signals of pMLC2 were amplified for 10 min and detected using the TSA Plus Fluorescein System (Perkin Elmer) by following the manufacture’s instruction. Nuclei were stained with Hoechst 33342 (1:5,000; Invitrogen).

In situ hybridizations were performed by adapting miRNA localization protocols to skin. Signals were detected using the TSA Plus Fluorescein System (Perkin Elmer) for fluorescent signal and posthybridization staining. The miRCURY LNA miR-205 detection probe (Exiqon) was 5′-DIG and 3′-DIG double labeled and hybridized to skin sections at 61 °C. Sections were stained with β4-integrin (BD Biosciences, 553745) and Alexa-Fluor-594-conjugated secondary antibody (1:2,000, Invitrogen/Molecular Probes, A11007) after developing.

Imaging was performed on a Leica DM5500B microscope with an attached Hamamatsu C10600-10B camera and MetaMorph (version 7.7; MDS Analytical Technologies) software, and Nikon W1 Dual CAM Spinning Disk at Northwestern University Feinberg School of Medicine.

### Single-Cell RNA-seq Assay and Data Analysis.

Single-cell RNA-seq was performed according to the manufacturer’s instruction (10× Genomics). Cells from both control and miR-205 inducible animals after 4-d induction were collected from the FACS sorting machine with cell surface proteins and H2b-GFP signals such that the epidermal cells, hair follicle cells, and dermal cells are in 3:5:2 ratio. Each sample was targeted to obtain ∼5,000 cells. Single-cell RNA-seq reads were sequenced on Illumina Hi-Seq 4000.

The Cell Ranger Single-Cell Software Suite was used to perform barcode processing and single-cell 3′ gene counting (https://support.10xgenomics.com/single-cell-gene-expression/software/overview/welcome). The barcodes, features, and matrix files were loaded into Seurat 3.0 for downstream analysis (https://satijalab.org/seurat/articles/install.html). For each sample, the analysis pipeline followed the guided tutorial. The cells were filtered with nFeature_RNA (>200 and <5,000) and mitochondrial percentage (<10). After clustering and UMAP dimension reduction, the cluster markers were used to identify distinct cell populations. For comparison among different samples, samples were integrated to promote the identification of common cell types and enable comparative analysis.

### Intravital Live Imaging and Image Processing and Quantification.

Intravital live imaging was performed as previously described (15, 51) with modifications. Mice used for imaging were sedated using 1% oxygen and ~2% isoflurane. Once a mouse was fully sedated, it was put on a warm pad at 37 °C. The oxygen and isoflurane were maintained during the course of imaging. Night-time ointment (Genteal, NDC 0078-0473-97) was applied to keep eyes moisturized. A custom-manufactured spatula was used to flatten the region of interest and maintained at adjustable height. Double-sided tapes were used to adhere the lower side of the ear onto the spatula. After applying long-lasting Genteal gel (0078-0429-47) to the region of interest, a second adjustable spatula, glued with cover-glass on one end, was gently pressed down to the ear so that the cover glass was right on top of the region. Long-lasting Genteal was applied on the cover-glass to keep the tip of the objective merged in during imaging. The Olympus FVMPE-RS multiphoton imaging system was applied for acquiring Krt14-H2b-GFP images. The lasers were Insight X3 with the wavelength set to 920 nm for GFP signals. The emission wavelength was 510 nm. For images, 10× and 25× objectives were used. The Leica DiveB Sp8 Multiphoton imaging system was used for E-cad-mCFP and mTmG/Krt14-H2B-GFP images. During the imaging session, the light should be turned off, and the stages and scope should be covered with a black curtain to avoid exposure to light. After the image session is done, the mouse was kept in oxygen to recover before sending back in cage. Two photon images were acquired using FluoView software from Olympus. The time-lapse images were aligned using Fiji > plugins > registration > descriptor-based series registration (2d/3d +t) before being exported. Then, images were exported to tif format using Fiji > plugins > bio-formats > bio-formats-exporter. The exported tif files were further converted to Imaris file format using Imaris File Converter software. The Imaris x64 9.7.2 were used for further analysis. The images were adjusted on x, y, or z plane for better visualization. Movies were also adjusted and generated from Imaris.

For HG volume quantifications, the 3D pictures were opened in Imaris and the epidermis and upper hair follicle regions were then cut off to acquire individual hair follicles, including bulge and HG. Individual HG regions were applied with the Imaris Surface module. The volume output of hair germ was used for quantification. The hair follicles with obvious drift were excluded to avoid artificial effects. Individual HG volumes are heterogeneous. To determine the dynamic changes of HGs at different stages and in different samples, we designated the largest value of the volume during a period of time as 100% and obtained relative percentage value at different time points by comparing with the largest value. The average cell volumes were obtained from total HG volume divided by cell numbers included for HG volume calculation.

## Statistics and Study Design.

In general, all sequencing experiments (RNA-seq) were repeated on at least two pairs of control and miR205 inducible per experiment. Single-cell RNA-seq was performed with one pair of control and miR-205-inducible samples at the same time on the same chip to avoid the batch effect. All experiments were designed such that there were always littermate controls. All statistical tests performed are as indicated in the figure legends. No statistical methods were used to predetermine the sample size. The experiments were not randomized, and the investigators were not blinded to allocation during experiments and outcome assessment, except where stated.

For all experiments with error bars, the SD was calculated to indicate the variation within each experiment. The numbers of animals used for phenotype study have been indicated in the manuscript and figure legends. Student’s *t*-test or the Mann–Whitney *U* test was used for most experiments, as indicated in figure legends.

## Supplementary Material

Appendix 01 (PDF)

Appendix 02 (PDF)

Dataset S01 (XLSX)

Dataset S02 (XLSX)

Movie S1. **Hair germ contraction during telogen**. In the 4-hour intravital imaging, the HG but not the bulge HF-SC compartment contracts by ~30% in volume. Note the constant position and location of each HF-SCs during the same period. All epithelial cells are labeled with H2b-GFP. The constant red dotted line outlines the HF-SC and HG compartments. The yellow dotted line and green arrowheads track the movement of the bottom of the HG.

Movie S2. **Dynamic contraction and enlargement activities of hair germ during telogen**. In the 6-hour intravital imaging, the HG exhibits pulsatile contraction and enlargement activities. Note the constant position and location of HF-SCs during the same period. All epithelial cells are labeled with H2b-GFP. The green arrowheads track the movement of the bottom of the HG.

Movie S3. **Hair germ contraction during telogen**. In the 4-hour intravital imaging, the HG but not the bulge HF-SC compartment contracts by ~30% in volume. The plasma membrane of epithelial cells is labeled with E-Cad-CFP. Note the strong E-Cad levels in the HF-SCs and the relatively weak E-Cad levels in the HG. The constant red dotted line outlines the HF-SC and HG compartments. The yellow dotted line and green arrowheads track the movement of the bottom of the HG.

Movie S4. **Hair germ enlargement during early anagen**. In the 4-hour intravital imaging, the HG enlarges by ~20% in volume in the absence of cell division. Note the HF-SC compartment also enlarges but less than the HG. All epithelial cells are labeled with H2b-GFP. The constant red dotted line outlines the HF-SC and HG compartments. The yellow dotted line and green arrowhead track the movement of the bottom of the HG.

Movie S5. **Hair germ enlargement in an miR-205 induced hair follicle**. In the 4-hour intravital imaging, the HG initially enlarges and then slightly contracts toward the end of the imaging session in the absence of cell division. Note the HF-SC compartment also enlarges slightly. All epithelial cells are labeled with H2b-GFP. The constant red dotted line outlines the HF-SC and HG compartments. The yellow dotted line and green arrowheads track the movement of the bottom of the HG.

## Data Availability

RNA-seq and single-cell RNA-seq data have been deposited in NCBI/GEO (GSE131205 (52) and GSE185117 (53)).
